# A case of coronary artery air embolism in a transplanted heart during cardiac allograft vasculopathy surveillance angiography

**DOI:** 10.21542/gcsp.2021.23

**Published:** 2021-10-30

**Authors:** Jeffrey F. Spindel, Vikas Singh, Mohammad Mathbout

**Affiliations:** 1 Department of Internal Medicine, University of Louisville School of Medicine, Louisville, Kentucky 40202, USA; 2 Division of Cardiovascular Medicine, Department of Medicine, University of Louisville School of Medicine, Louisville, Kentucky 40202, USA

## Abstract

Coronary air embolism is a rare iatrogenic complication during invasive coronary angiography or angioplasty that can cause acute chest pain, hypotension, ST-segment elevation myocardial infarction, and even death. We present a case of left anterior descending coronary artery air embolization in a 58-year-old heart transplant patient that occurred during cardiac allograft vasculopathy surveillance angiography. The patient was managed successfully with rapid coronary injections of heparinized saline, catheter disengagement to increase coronary blood flow, and supplementation of 100% oxygen to dissolve the coronary air embolus. This case highlights this rare complication of coronary angiography, importance of prompt recognition of the pathology and subsequent management.

## Case report

**Figure 1.**
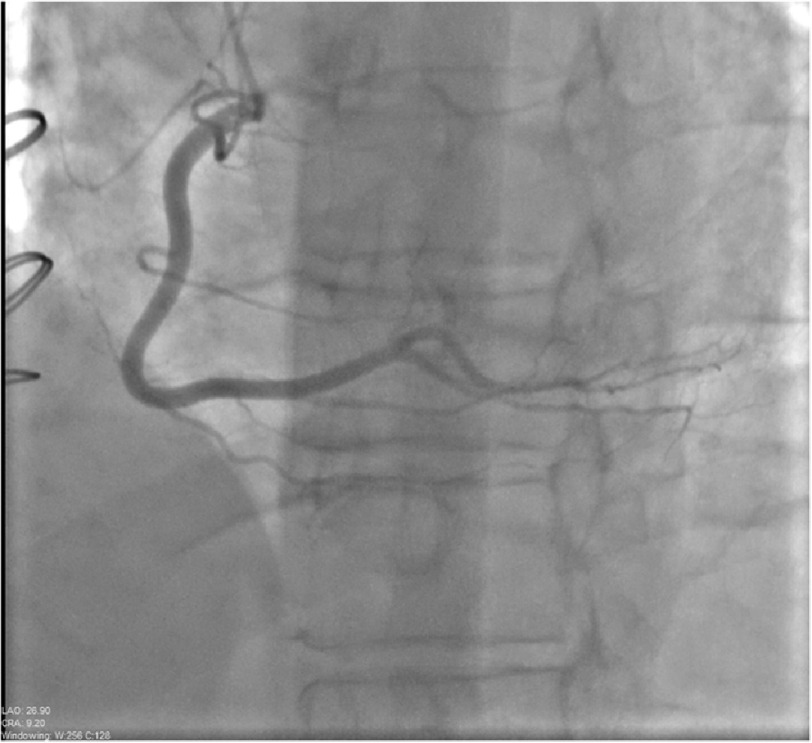
A 5 Fr Judkins right 4 catheter was used for angiography of the right system demonstrating no luminal disease (image obtained in LAO/Cranial view).

**Figure 2.**
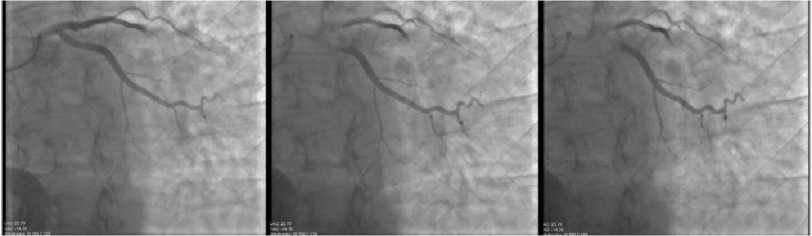
Image sequence shows a normal left circumflex artery, a missing middle segment of the LAD, and an oscillating coronary embolism with a back-and-forth movement, causing 100% obstruction in the mid-distal left anterior descending artery, TIMI-0 flow (image sequence obtained in LAO/Caudal view).

A 58-year-old female with past medical history of heart transplant 5 years prior (due to non-ischemic cardiomyopathy) presented for yearly screening of cardiac allograft vasculopathy (CAV) with left heart catheterization (LHC) and coronary angiography. Percutaneous access was performed through the right radial artery. A 5-Fr Judkins right 4 catheter was used for angiography of the right system and no abnormalities were noted (see Figure 1). A Judkins Left 3.5 catheter was used for the left system. Coronary angiography of the left main artery revealed a normal left circumflex artery and a missing middle segment of the LAD. Further imaging was concerning for a coronary air embolism (CAE), oscillating with a back-and-forth movement, causing 100% flow obstruction in the mid-distal left anterior descending artery (TIMI-0) as seen in Figure 2.

**Figure 3.**
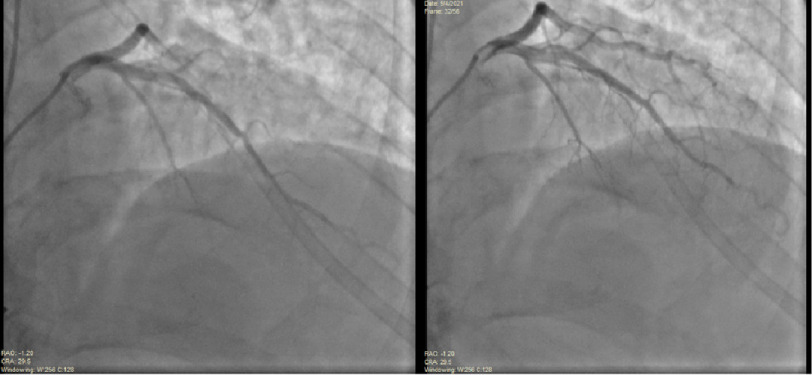
Repeat angiography of the left coronary system demonstrated restoration of TIMI-3 blood flow into the LAD (image sequence obtained in AP/Cranial view).

**Figure 4.**
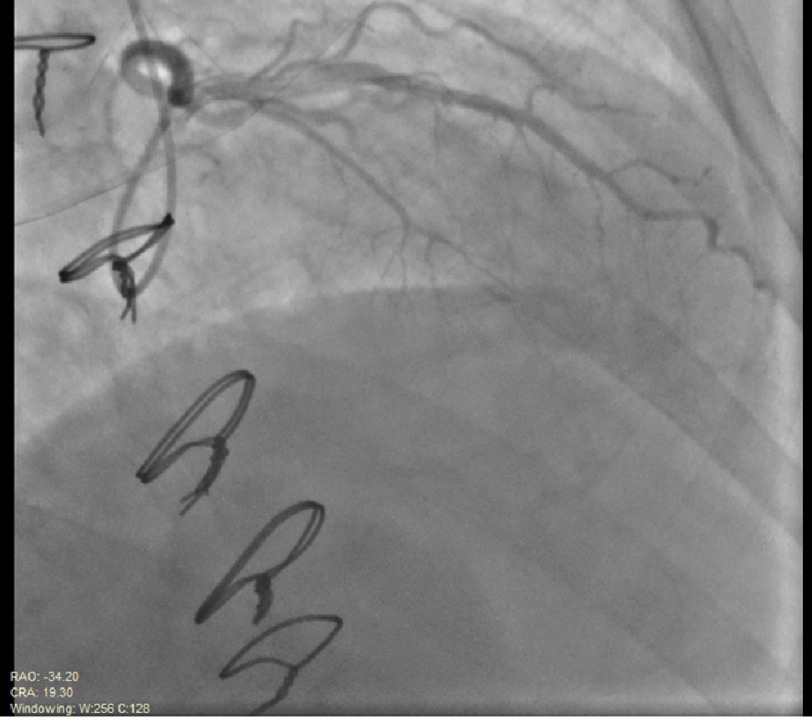
Repeat angiography of the left coronary system demonstrated resolution of the coronary embolism and restoration of TIMI-3 blood flow into the LAD. Relative Visipaque contrast streaming is attributed to the hyperdynamic nature of flow immediately post revascularization (image sequence obtained in RAO/Cranial view).

Simultaneously, the patient had symptoms of chest pressure, diaphoresis, and nausea. Marked ST segment elevations were noted on the cardiac monitor. Rapid injections of heparinized saline into the LAD were performed. The guide was then immediately disengaged from the left main coronary artery ostium, allowing more blood flow. The patient was placed on 100% oxygen. Within 4 min of oxygen supplementation, the ST changes and symptoms resolved. Follow-up angiography of the left coronary system was performed showing restoration of TIMI-3 blood flow into the LAD and demonstrating no luminal disease (Figure 3, 4).

Post procedure transthoracic echocardiogram revealed normal left ventricular function with an ejection fraction of 62%. No changes from prior echocardiographs were noted. The patient was discharged home the same day with a plan to resume routine CAV surveillance schedule.

## Discussion

Coronary air embolism (CAE) is a rare iatrogenic complication during invasive coronary angiography or angioplasty that can cause acute chest pain, hypotension, ST-segment elevation myocardial infarction, and death^1^. CAE was reported to occur in only 0.27% of procedures, though this may be less in the modern era of invasive cardiology, and usually occurs in a single vessel, affecting the LAD most^1,2^. CAE most commonly occurs during introduction, removal, or exchange of equipment, including the guide wire, balloon, or stents, and is usually due to inadequate aspiration of equipment^1^.

Small air emboli may self-resolve, however larger emboli may not, as the time for reabsorption depends on size, partial pressures of the gases in and around the embolism, and tissue perfusion, which is reduced by the embolism^3^. Management is first aimed at promoting nitrogen diffusion *via* use 100% inhaled oxygen^1,2^. Aspiration using thrombectomy catheters has been performed successfully, but may not result in complete resolution^2,4–6^. Other treatment strategies include increasing coronary arterial pressures using inotropes or an intra-aortic balloon pump, dissipation of the embolism using injected saline or blood, or vasodilation with adenosine, calcium channel blockers, or nitrates to treat coronary slow flow^1,4^.

In our case, catheter disengagement and prompt oxygen supplementation were adequate in dissolving an air embolus causing 100% blockage of the mid-LAD. Oxygen supplementation increases the partial pressure (PaO_2_) in the blood, leading to an increase in tissue oxygen concentration with a corresponding decrease in nitrogen concentration. The decrease in tissue nitrogen content leads to a favorable diffusion gradient out of the nitrogen-rich air embolus^3,6^. After identification of a CAE, supplementation with 100% oxygen should be performed promptly, regardless of the use of further treatment strategies.

## What have we learned?

• Coronary air embolism is a rare complication during invasive coronary angiography or angioplasty that can cause acute chest pain, hypotension, ST-segment elevation myocardial infarction, and death.
• Coronary air embolism most commonly occurs during introduction, removal, or exchange of equipment, usually due to inadequate aspiration of equipment prior to introduction.
• Management is aimed at promoting nitrogen diffusion with 100% inhaled oxygen, aspiration or dissipation of the embolism, increasing coronary arterial perfusion, or vasodilation to treat coronary slow flow.

### Author Statement

**Conceptualization:** Vikas Singh, Mohammad Mathbout

**Writing –Original Draft Preparation:** Jeffrey F. Spindel, Mohammad Mathbout

**Writing –Reviewing & Editing:** Jeffrey F. Spindel, Mohammad Mathbout, Vikas Singh

## References

[1] Kariyanna PT, Jayarangaiah A, Jayarangaiah A, Hegde Sudhanva, Marmur Jonathan D, Haseeb Syed, Song Teresa, Singh Navneet, McFarlane Samy I (2018). Coronary air embolism during coronary angiography: A systematic review. SciFed J Cardiol.

[2] Khan M, Schmidt DH, Bajwa T, Shalev Y (1995). Coronary air embolism: incidence, severity, and suggested approaches to treatment. Cathet Cardiovasc Diagn.

[3] Hlastala MP, Van Liew HD (1975). Absorption of in vivo inert gas bubbles. Respir Physiol.

[4] Yew KL, Razali F (2015). Massive coronary air embolism successfully treated with intracoronary catheter aspiration and intracoronary adenosine. Int J Cardiol.

[5] Solodky A, Birnbaum Y, Assali A, BenGal T, Strasberg B, Herz I (2000). Coronary air embolism treated by bubble aspiration. Catheter Cardiovasc Interv Off J Soc Card Angiogr Interv.

[6] Dib J, Boyle AJ, Chan M, Resar JR (2006). Coronary air embolism: A case report and review of the literature. Catheter Cardiovasc Interv.

